# Milk- and wheat protein drinks for double blind placebo controlled food challenge in adults: a simple tool for diagnosis

**DOI:** 10.1186/s13601-019-0284-5

**Published:** 2019-09-18

**Authors:** J. van Odijk, H. M. Lindqvist

**Affiliations:** 1 Dept of Respiratory Medicine and Allergology, Sahlgrenska University Hospital, Gothenburg, Sweden; 2 Dept. of Internal Medicine and Clinical Nutrition, Sahlgrenska Academy at Göteborg University, Gothenburg, Sweden

**Keywords:** DBPCFC, Food hypersensitivity, FODMAP, Sensory test, Triangle test

## Introduction

Increasing number of adults report food allergies, where > 50% express gastrointestinal symptoms after ingestion of certain foods [1]. Diagnosing diffuse gastrointestinal problems or hypersensitivity to certain foods often requires oral food challenges, especially when patient history is vague and/or clinical tests are inconclusive. Double blind placebo controlled food challenge (DBPCFC) is considered the golden standard model [2], but requires that the food tested is blinded i.e. differences between active and placebo products can’t be detected by taste, smell or texture. To prove this, sensory testing is needed. The most frequently used model is the triangle test where the purpose is to identify one odd sample regardless of type of difference identified [3]. However, most DBPCFC recipes have been validated within pediatric populations [4] and may not be optimal for adults, since higher doses normally are needed to provoke symptoms. Few studies have tested larger volumes for oral challenges or are not based on ordinary staple foods.

Food hypersensitivity diagnose and irritable bowel syndrome (IBS) have been suggested to overlap in adult patients [5]. Although it has not been confirmed that food intolerance (self-reported) is related to IBS symptom severity, a low content of fermentable carbohydrates, as FODMAP, is preferable to avoid possible gastrointestinal symptoms caused by these [5, 6].

### Aim

To develop simple, validated recipes for DBPCFC drinks, based on ordinary staple foods, to be used as support for diagnosis of milk- and wheat protein hypersensitivity in adults.

## Method

### DBPCFC drinks

The validated model for DBPCFC used at the clinic is based on serving one challenge dose per occasion and was set as a Ref. [7]. Thus, a goal of hiding 20 g of wheat flour, comparable to 1 slice of bread (i.e. 1.7 g wheat protein) and 100 ml of milk (i.e. 3 g milk protein) was set. Staple foods available in ordinary grocery stores were tested in challenge drinks in different amounts to mimic a smoothie drink. Every challenge contains two or three active doses in a set of five. Rice-, coconut- and oatmeal drinks were tested as liquid vehicles. For flavouring and to mask taste and structure, orange juice, fruit purée cocoa powder and vanilla extract were tested. If a test drink did not pass either an acceptance test as first step or the triangle test as second step, the recipe was further developed. Unsuccessful recipes are not presented.

### Calculation of fermentable carbohydrates

The FODMAP content was calculated for all the drinks using a special version of the software Dietist XP 3.1 (kostdata.se, Stockholm, Sweden). The software is linked to a food composition table provided by the National Food Agency in Sweden, and to a Swedish database with FODMAP content developed at Sahlgrenska University Hospital. The FODMAP database contained information about fructose, fructan, lactose, galacto-oligosaccharide (GOS) and polyol content (g/100 g) from published sources [8].

### Triangle test

Students and staff at Sahlgrenska Academy, Gothenburg University and Sahlgrenska University Hospital were invited to participate to evaluate sensory differences between the drinks. The inclusion criteria were: > 18 years of age, non-smokers, no food allergy to any of the content. Subjects with an ongoing cold were excluded. All subjects participated anonymously and no biological material was collected. A minimum number of panelists per test was set according to the literature [3].

Two different occasions were used for testing wheat (total n = 73) and milk (total n = 61) drinks, respectively, to ensure that the instruction was sufficient independent of who prepared them and to strengthen the triangle test. The samples were served in open cups, each containing 40–50 ml of the sample. The panelists were served three samples using the following combinations: (AAP, APA, PPA, PAP, APP, PAA etc.). The panelists were instructed to test each sample once only and to drink water between tests. If no different sample was detected, they were asked to guess.

### Statistics

Triangle test significance was calculated based on ${\text{x}} = 0.4717 \times {\text{z }} \times \sqrt n + \left[ {\left( {2n + 3} \right)/6} \right],$ where x is the minimal number of correct answers to detect a difference, n = number of panelists, z is 1.64 for *p* value 0.05. The test was regarded as not significant, if the number of correct answers were below the minimal number of correct answers to detect a difference (x), with a p-value set at 0.05 significance.

## Results

The final drink recipes are presented in Table 1. The calculated content of FODMAP in the challenge drinks were low, where only FOS and GOS could be detected in the active wheat challenge drink and none of the other drinks contained any fermentable carbohydrates at all. In the repeated test 42% and 39% respectively selected the correct sample in the wheat- and milk triangle tests (Table 2). Both tests had less correct answers than expected if a true sensory difference exists (p < 0.05).

**Table 1:** DBPCFC recipes for challenge drinks, including content of fermentable carbohydrates

Ingredient	Active drink (g)	Placebo drink (g)	FOS active (g)	GOS active (g)	FOS placebo (g)	GOS placebo (g)
Wheat recipes						
Coconut drink^a^	225	225	–	–	–	–
Drinking chocolate powder^b^	12	13.5	–	–	–	–
Heat treated flour^c^	25		0.38	0.15	–	–
Gluten-free mix^d^		17	–	–	–	–
Vanilla sugar	3	3.5	–	–	–	–
Energy (kcal)	197	169				
Milk recipes						
Coconut drink^a^	100	200	–	–	–	–
Drinking chocolate powder^b^	20	20	–	–	–	–
Lactose free skimmed milk	100		–	–	–	–
Vanilla sugar	3	3	–	–	–	–
Energy (kcal)	142	132				

*g* gram, *FOS* fructo-oligo saccharides, *GOS* galacto-oligo saccharides

^a^Alpro soy drink, original, no added sugar

^b^Oboy chocolate drinking powder: sugar, 18% fat reduced cocoa, soy lecithin, dextrose, salt, vanilla

^c^Kungsörnen ideal wheat flour. 100% heat treated flour

^d^Semper fine gluten-free mix: maize starch, rice flour, potato flour, modified potato starch, sugar

**Table 2:** Evaluation of triangle test for identification of active food sample

	No. of panelists	Mean age (range)	Men/women	Correct answer	Critical value	p-value^a^
Wheat recipe (occasion 1)	38	22 (19–31)	14/24	16	18	> 0.05
Wheat recipe repeated (occasion 2)	35	25 (19–39)	4/31	15	17	> 0.05
Milk recipe (occasion 1)	43	28 (20–52)	9/34	17	20	> 0.05
Milk recipe repeated (occasion 2)	18	26 (19–41)	3/15	9	10	> 0.05

^a^Significance is determined by a triangle test table where the number of correct answers must be higher than a critical value specific for the number of participants and therefore no exact p-values are calculated

## Discussion

We have shown that it is possible to hide an adult portion of milk or wheat, respectively, in a challenge drink based on ordinary staple foods and with low content of fermentable carbohydrates. Since there is an increased focus on the role of fructans and gluten hypersensitivity in the diet of IBS patients [9], we believe that this type of food challenge drinks may be useful for the testing of a broad spectrum of food hypersensitivity. To our knowledge, no other recipes for challenge drinks are available that present fermentable carbohydrates content.

In addition the developed recipes are cheap, easy to prepare and ingredients are found in grocery stores. Although several other flavors were tested, chocolate and vanilla were the most accepted and best masking other flavors, in line with previous studies [10]. For the wheat recipe, standard heat treated wheat flour was tested against gluten free flour. It also contains larger particles that are more easily liquid soluble and more similar in form to wheat normally consumed.

A limitation of the study is the use of volunteer panels, which has been connected to overestimated blinding results [11]. To avoid this, we have performed repeated triangle tests with different taste panels including mainly young women and only healthy, non-smoking, non-allergic volunteers, in separate settings to strengthen the results.

In conclusion: this study has shown that is possible to hide amounts comparable to adults portions of wheat and milk protein in challenge drinks that can be used in double blind placebo controlled food challenges.

## Data Availability

Data available from corresponding author.
